# Comparative analysis of nitrogen content and its influence on actinorhizal nodule and rhizospheric microorganism diversity in three *Alnus* species

**DOI:** 10.3389/fmicb.2023.1230170

**Published:** 2023-12-15

**Authors:** Yuwei Yuan, Zhi Chen, Xin Huang, Fang Wang, Hongying Guo, Zhen Huang, Hanbo Yang

**Affiliations:** ^1^Forestry Ecological Engineering in the Upper Reaches of the Yangtze River Key Laboratory of Sichuan Province, National Forestry and Grassland Administration Key Laboratory of Forest Resource Conservation and Ecological Safety on the Upper Reaches of the Yangtze River, Rainy Area of West China Plantation Ecosystem Permanent Scientific Research Base, College of Forestry, Sichuan Agricultural University, Chengdu, China; ^2^Sichuan Key Laboratory of Ecological Restoration and Conservation for Forest and Wetland, Sichuan Academy of Forestry, Chengdu, China; ^3^Sichuan Academy of Grassland Sciences, Chengdu, China

**Keywords:** *Alnus*, *Frankia*, nitrogen nutrients, 16S rRNA, *nifH*, nitrogen fixation

## Abstract

*Alnus* spp. (alder) are typical nonleguminous nitrogen-fixing trees that have a symbiotic relationship with *Frankia*. To explore the differences in nitrogen-fixing microorganisms between three alders (*A. cremastogyne*, *A. glutinosa*, and *A. formosana*) with different chromosome ploidies, the community structure and compositional diversity of potential nitrogen-fixing microorganism in root nodules and rhizosphere soil were comparatively analyzed using 16S rRNA and nitrogenase (*nifH*) gene sequencing. The nitrogen contents in the root nodules and rhizosphere soil were also determined. The results showed that the contents of total nitrogen and nitrate nitrogen in the root nodules of the three alders are significantly higher than those in the rhizosphere soils, while the ammonium nitrogen content show the opposite trend. The family, genus, and species levels showed obviously differences between root nodules and rhizosphere soils, while there were no significant differences at the classification level between the three alders. At the phylum level, the dominant phyla from 16S rRNA and *nifH* gene data in the root nodules and rhizosphere soil of the three alders are phylum Actinomycetota and phylum Pseudomonadota, respectively. The LEfSe results showed that there are significant differences in the dominant groups in the root nodules and rhizosphere oil of the three alders. The relative abundances of dominant groups also showed obvious differences between the root nodules and rhizosphere soils of three alders. The relative abundances of *Frankia* and unclassified_*Frankia* in root nodules are obviously higher than those in rhizosphere soils, and their relative abundances in *A. glutinosa* root nodules are significantly higher than those in *A. cremastogyne* and *A. formosana* at the genus and species levels. The diversity of potential nitrogen-fixing microorganism from 16S rRNA and *nifH* gene data in the *A. glutinosa* root nodules and rhizosphere soils are all higher than those in *A. cremastogyne* and *A. formosana*. The results of functional prediction also showed that the OTUs for nitrogen fixation, nitrate respiration, and ureolysis in *A. glutinosa* root nodules are higher than those in the other two alders. Redundancy analysis revealed that the total nitrogen content mostly affects the *Frankia* community. Overall, there are significant differences in the community composition and structure of potential nitrogen-fixing microorganism in the root nodules and rhizosphere soils between the three alders. *A. glutinosa* showed a relatively stronger nitrogen fixation capacity than *A. formosana* and *A. cremastogyne*. The results help elucidates how the community structure and nitrogen-fixing ability of potential nitrogen-fixing microorganism differ between alder species and serve as a reference for applying *Frankia* to alder plantations.

## 1 Introduction

Biological nitrogen fixation (BNF) is a microbial-mediated process based on nitrogen-fixing enzymes that convert atmospheric nitrogen (N_2_) into an ammonium form (NH_3_) that is easily taken up by plant roots (Soumare et al., 2020). This is important for promoting plant growth and development, reducing nitrogen (N) fertilizer application, and enhancing soil fertility (Soumare et al., 2020; Aasfar et al., 2021). This symbiosis between plants and diazotrophic soil bacteria is found in a very limited number of plants, with two types of bacteria, *Rhizobium* and *Frankia*, defining legume-*Rhizobium* symbiosis and plant-*Frankia* symbiosis, respectively (Kim Tiam et al., 2023). These microorganisms include the nonleguminous *Parasponia* species (family Cannabaceae) and *Frankia* sp. (gram-positive) members of the Actinomycetes family that associate with a broad spectrum of plants belonging to eight families collectively called actinorhizal plants (Santi et al., 2013). Actinorhizal plants are woody shrubs and trees, except for the genus *Datisca*, which is herbaceous (Benson et al., 2004; Ardley and Sprent, 2021). At present, over 200 strains of *Frankia* have been isolated from many, although not all, actinorhizal plant species (Santi et al., 2013). Previous studies have shown that the inoculation of *Frankia* strains is an appropriate strategy to enhance *Frankia*-*Alnus* symbiosis, resulting in increased plant growth performance and nitrogen availability (Bernie Steele et al., 1989; Nickel et al., 2001). In pristine soils, the rates of nitrogen fixation in actinorhizal alders are known to be comparable to those in legumes; alfalfa and clover can fix 57–300 kg Nꞏha^−1^ꞏyear^−1^ and 104–160 kg Nꞏha^−1^ꞏyear^−1^, respectively, while black, red, and sitka alders can fix nitrogen in the range of 40–300 kgꞏha^−1^ꞏyear^−1^ (Hibbs and Cromack Jr, 1990; Zubberer, 2005; Roy et al., 2007). Previous studies have shown that the inoculation of *Frankia* strains is an appropriate strategy to enhance *Frankia*-*Alnus* symbiosis, resulting in increased plant growth performance and nitrogen availability (Bernie Steele et al., 1989; Nickel et al., 2001). In pristine soils, the rates of nitrogen fixation in actinorhizal alders are known to be comparable to those in legumes; alfalfa and clover can fix 57–300 kg Nꞏha^−1^ꞏyear^−1^ and 104–160 kg Nꞏha^−1^ꞏyear^−1^, respectively, while black, red, and sitka alders can fix nitrogen in the range of 40–300 kgꞏha^−1^ꞏyear^−1^ (Hibbs and Cromack, 1990; Zubberer, 2005; Roy et al., 2007). In addition, actinorhizal plants can regulate N fixation in response to N status, but compared to legumes, actinorhizal fixation is less variable and remains at a high level within the soil N supply range (Ardley and Sprent, 2021). Plant growth, biomass, aboveground and root N contents, and survival rate after field transplantation may be greatly enhanced by the symbiotic relationship between *Frankia* and actinorhizal plants (Diagne et al., 2013). In addition, it is possible to alleviate the adverse effects of the abiotic and biotic pressures that result in land degradation using actinorhizal plants (Diagne et al., 2013). In particular, to overcome the problem of insufficient fertility of degraded soil in tropical countries, fast-growing nitrogen-fixing trees, such as actinorhizal trees, can be used in combination with biofertilization (Diagne et al., 2013). *Frankia*-inoculated trees not only have increased nitrogen nutrition but also have increased access to soil phosphorus (Chen et al., 2022). In forestry production, these woody nitrogen-fixing species are one of the major sources of biologically fixed atmospheric N due to their widespread distribution, great adaptability, and ability to enhance soil fertility. Thus, *Frankia*-inoculated trees are pioneer trees for greening barren mountains, are a significant N supplier in forest ecosystems and have crucial scientific significance and application value (Peng, 2008).

There are various types of symbiotic nitrogen-fixing bacteria, that differ greatly due to differences in tree species and soil. Traditional research on nitrogen-fixing bacteria in plants is conducted through the methods of pure culture and isolation, but due to the limitations of culture conditions, unculturable bacteria usually account for a large proportion of the microbiome. Therefore, the results of traditional pure culture analysis often do not fully reflect the real composition of microbial species in the sample. With the rapid development of high-throughput sequencing technology, the limitations of microbiology based on traditional pure culture can be overcome, and the dominant microflora in the sample can be determined, which can more accurately reflect the microbial community structure in the sample (Rau et al., 2015). In the taxonomy of the genus *Frankia*, Ghodhbane-Gtari et al. (2010) identified the genus *Frankia* in the order Actinomycetales based on the results of phylogenetic analysis using 16S rRNA sequencing (Ghodhbane-Gtari et al., 2010). Then, 16S-23S rRNA internal transcribed spacer sequences were sequenced from 53 *Frankia* strains, indicating that comparative analyses of the 16S-23S rRNA intergenic spacer region of *Frankia* strains were not useful in assigning them to their respective cluster or host infection group. Later, Gtari et al. (2019) provided an update of the taxonomy of *Frankia* based on the integration of genomic data into the polyphasic taxonomy approach, enabling valid naming of several *Frankia* species (Gtari et al., 2019). Additionally, Gtari (2022) reclassified the *Frankia* genus into four separate genera by elevating each of the four clusters to the rank of genus. In addition to *Frankia*, three new genera were introduced: *Protofrankia* (strains that infect *Coriariaceae*, *Datiscadeae*, *Dryadoideae*, and *Ceanothus*), *Parafrankia* (*Elaeagnaceae*, *Colletieae*, *Morella*, and *Gynmnostoma*), and *Pseudofrankia* (unable to fix nitrogen and/or to reinfect their hos plants) (Gtari, 2022). The *nifH* gene encodes nitrogenase ferritin and is the most conserved functional gene contained by all nitrogen-fixing microorganisms (Hennecke et al., 1985; Haukka et al., 1998). Therefore, the *nifH* gene is the biomarker most widely used to study the ecology and evolution of nitrogen-fixing bacteria (Gaby and Buckley, 2014). For instance, Lin et al. (2018) evaluated whether long-term fertilization affected the abundance, diversity, and community structure of nitrogen-fixing bacteria using sequencing of *nifH* functional genes of the microbiome (Lin et al., 2018). In turn, Groß et al. (2022) proposed that biological N fixation is a ubiquitous microbial process in the deadwood of native European tree species with the help of *nifH* gene sequencing (Groß et al., 2022). Therefore, 16S rRNA and *nifH* gene sequencing is a very efficient and accurate method to study the community structure and diversity of microorganisms related to nitrogen fixation.

*Alnus* spp. (alder) is the most widely distributed plant genus of actinorhizal plants and is the dominant host of *Frankia* in Northern Hemisphere temperate forests (Perakis and Pett-Ridge, 2019; Markham and Anderson, 2021). Moreover, *Alnus* is the only nitrogen-fixing tree genus in Betulaceae that can form symbioses with *Frankia* (Benson and Silvester, 1993; Guo et al., 2019). Although the amount of fixed N transported by actinorhizal alder to nearby soils varies greatly (40–300 kg Nꞏha^−1^ꞏyear^−1^), alder is known to significantly contribute to global N fixation (Roy et al., 2007). Moreover, alder can grow in severe conditions with low soil nutrients due to their symbiotic N fixation, and *Alnus* spp. are important in the dynamic succession and nutrient cycle of many ecosystems (Roy et al., 2007; Kennedy et al., 2010). At present, research on alder symbiotic N fixation focuses mostly on the diversity of and symbiotic relationship with nitrogen-fixing bacteria in root nodules (Balkan et al., 2020; Wolfe et al., 2022; Vemulapally et al., 2022a), while research on the actinobacteria and *Frankia* found in the root nodules of varied ploidy *Alnus* spp. has fallen behind in terms of community composition, structural diversity, and N fixation capacity. Furthermore, it has been shown that root nodule formation is not a function of the relative abundance or functional diversity of specific *Frankia* in the soil; instead, plants select *Frankia* from the soil to form root nodules (Ben Tekaya et al., 2018; Vemulapally et al., 2022a). Therefore, in this study, the root nodules and rhizosphere soils of three alders with different ploidies (*A. formosana*, 2n = 56, *A. cremastogyne*, 2n = 56, *A. glutinosa*, 2n = 28) (Han-bo et al., 2013; Longbing et al., 2013) were chosen, and their N nutrients and potential nitrogen-fixing microorganism communities were compared through 16S rRNA and *nifH* gene sequencing. This study provides a theoretical reference for the diversity of microorganisms in nonleguminous nitrogen-fixing woody plants, the selection of tree species with high nitrogen-fixing ability and the symbiosis between nitrogen-fixing *Frankia* and *Alnus* spp.

## 2 Materials and methods

### 2.1 Experimental design

The seeds of *Alnus glutinosa* (2n = 28), *A. formosana* (2n = 56), and *A. cremastogyne* (2n = 56) were sown at the nursery in Tangchang city, Sichuan Province, China in March 2021 (Han-bo et al., 2013; Longbing et al., 2013). Three consistently growing seedlings per alder (three biological replicates) were selected in the current year and transplanted to containers with light substrate (sterilized perlite: sterilized vegetable garden soil = 1:5). Then, the container seedlings were transferred to greenhouse cultivation. The root nodule and rhizosphere soil were sampled when the seedlings were 2-years old. The sampling tools (scissors, tweezers, etc.) were rinsed with 90% ethyl alcohol to prevent cross-contamination. The bulk soils were shaken off on an ultraclean bench, and then the rhizosphere soils (the soil attached to the root approximately 1 mm thick) from *A. glutinosa*, *A. formosana*, and *A. cremastogyne* (named AG_S, AF_S, and AC_S, respectively) were sampled and stored at 4°C until they were processed and analyzed for N determination. The roots and nodules were washed with deionized water to remove soil and organic matter. Then, all the root nodules per seedling from *A. glutinosa*, *A. formosana*, and *A. cremastogyne* (named AG_RN, AF_RN, and AC_RN, respectively) were cut by scissors, rinsed with 90% ethyl alcohol, and placed into sterile centrifuge tubes using tweezers. Finally, the root nodules were washed with 0.6% hypochlorite three times to remove the interference of other microorganisms on the surface of the nodules.

### 2.2 DNA extraction

DNA was extracted with the TGuide S96 Magnetic Soil/Stool DNA Kit (Tiangen Biotech (Beijing) Co., Ltd.) according to the manufacturer’s instructions. The Qubit dsDNA HS Assay Kit and Qubit 4.0 Fluorometer (Invitrogen, Thermo Fisher Scientific, Oregon, USA) was used to determine the DNA concentration in the samples.

### 2.3 Amplicon sequencing

The 16S rRNA gene and *nifH* gene from the genomic DNA extracted from each sample were amplified using nested PCR primers (243F, A3R: GGATGAGCCCGCGGCCTA, CCAGCCCCACCTTCGAC; 341F, 805R: CCTACGGGNGGCWGCAG, GACTACHVGGGTATCTAATCC) and *nifH* primers (F: TGYGAYCCNAARGCNGA and R: ADNGCCATCATYTCNCC). For deep sequencing, sample-specific Illumina index sequences were added to the tails of the forward and reverse 16S primers and *nifH* primers. DNA template 5–50 ng, primers (10 mM) 0.3 μL, KOD FX Neo Buffer 5 μL, dNTP (2 mM each) 2 μL, KOD FX Neo 0.2 μL, and ddH2O up to 10 μL were used in the PCR. Following a preliminary step of initial denaturation at 95°C for 5 min, there were 25 cycles of denaturation at 95°C for 30 s, annealing at 50°C for 30 s, and extension at 72°C for 40 s, followed by a final step at 72°C for 7 min for 16S rRNA amplification. For the *nifH* gene, the amplification program was as follows: 95°C for 5 min; 10 cycles of 95°C for 45 s, 65°C for 45 s, and 72°C for 60 s; 30 cycles of 95°C for 45 s, 56°C for 45 s, and 72°C for 60 s; and a final step at 72°C for 7 min. Agencourt AMPure XP Beads (Beckman Coulter, Indianapolis, IN) were used to purify the total amount of PCR amplicons, and the Qubit dsDNA HS Assay Kit and Qubit 4.0 Fluorometer (Invitrogen, Thermo Fisher Scientific, Oregon, USA) was used to quantify the results. Amplicons were pooled in equal amounts following the individual quantification step. The Illumina NovaSeq 6000 (Illumina, Santiago, CA, USA) was used for sequencing of the built-in library.

### 2.4 Bioinformatics analysis

BMK Cloud (Biomarker Technologies Co., Ltd., Beijing, China) was used in the bioinformatics analysis. Trimmomatic v0.33 (Edgar, 2013) was used to filter raw data primarily based on the quality of a single nucleotide. Using Cutadapt v1.9.1 (Callahan et al., 2016), primer sequences were identified and removed, which finally generated high-quality reads without primer sequences. The clean reads obtained from previous steps were assembled by USEARCH v10.0 (Segata et al., 2011), followed by denoising and chimera removal using dada2 (Callahan et al., 2016) and UCHIME v8.1 (Quast et al., 2012). The high-quality nonchimeric reads generated from the above steps were used in the following analysis. Using USEARCH v10.0 (Edgar, 2013), sequences with 97% similarity were clustered into the same operational taxonomic unit (OTU), and OTUs with a relative abundance <0.005% were filtered. For 16S rRNA sequencing analysis, with a confidence threshold of 70%, taxonomy annotation of the OTUs was carried out with the SILVA database (Quast et al., 2012) and the naïve Bayes classifier. For *nifH* gene sequencing analysis, taxonomic annotation of the OTUs was carried out with the FunGene database (Fish et al., 2013). QIIME2 (Bolyen et al., 2019) and R applications (R Core Team, 2022) were used to calculate and display the alpha diversity, respectively. Beta diversity was also calculated by QIIME2 (Bolyen et al., 2019) to assess how similar microbial communities from various samples were to one another. To examine beta diversity, nonmetric multidimensional scaling (NMDS) was employed (Looft et al., 2012). Additionally, we used linear discriminant analysis (LDA) effect size (LEfSe) (Segata et al., 2011) to test whether there were any groups with significantly different taxa. The cutoff for discriminative features was set at a logarithmic LDA score of 2.0. Redundancy analysis (RDA) was carried out in R using the package “vegan” (Oksanen et al., 2022) to investigate the differences between the microbiota and other variables.

### 2.5 Nitrogen determination

The cleaned root nodules were crushed at 105°C for 30 min and dried at 70°C to a constant weight. The rhizosphere soil was air dried after removing plant and animal residues, stones, and other debris. Then, the cleaned rhizosphere soil was ground and screened with a sieve net (0.25 mm and 1 mm) to determine the content of nitrogen types. The content of total nitrogen (TN) was determined by the Kjeldahl method, and those of nitrate nitrogen (NN) and ammonium nitrogen (AN) were determined by colorimetry through standard soil and plant physical and chemical analyses (Lu, 1999).

### 2.6 Statistical analysis

Alpha diversity indices (Chao1, Ace, Shannon, Simpson, Coverage, and PD_whole_tree) of actinobacteria and nitrogen-fixing bacteria in the root nodules and rhizosphere soils were estimated using QIIME2 (Bolyen et al., 2019). NMDS was carried out using QIIME2 (Bolyen et al., 2019), beta diversity analysis was conducted based on unweighted independent OTUs (Jaccard), and the distance algorithm used to compare the diversity of actinomyces and N-fixing bacteria in the root nodules and rhizosphere soils was binary_jaccard. LEfSe was performed to analyze the differences in actinobacteria and N-fixing bacteria between the three alder species with an LDA threshold of 2.0 at the taxonomic level from phylum to species. The functions of actinobacteria in the root nodules and rhizosphere soils were predicted using PICRUSt2 (Langille et al., 2013). One-way analysis of variance (ANOVA) (*p* < 0.05) and multiple comparisons (Duncan, *α* = 0.05) of TN, NN, and AN in the root nodules and rhizosphere soils among the three alders were performed in R.

## 3 Results

### 3.1 Nitrogen nutrient characteristics of root nodules and rhizosphere soil

**Figure 1:**
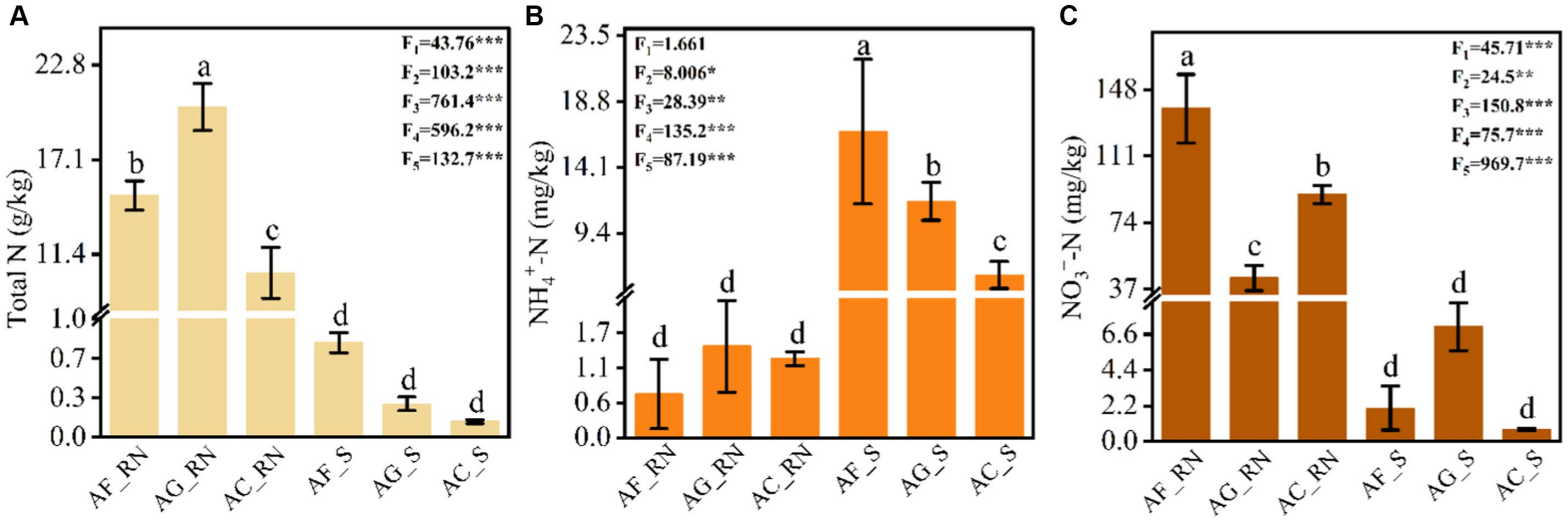
Contents of total nitrogen **(A)**, ammonium nitrogen **(B)**, and nitrate nitrogen **(C)** in the root nodules and rhizosphere soils of three alder species. AF_RN, AG_RN, and AC_RN represent the root nodules of *A. formosana*, *A. glutinosa*, and *A. cremastogyne*, and AF_S, AG_S, and AC_S represent the rhizosphere soil of *A. formosana*, *A. glutinosa*, and *A. cremastogyne*, respectively. F_1_ and F_2_ correspond to the *F* value of ANOVA of nitrogen nutrients among root nodules and rhizosphere soils of the three alders, respectively. F_3_–F_5_ correspond to the *F* value of ANOVA of nitrogen components among root nodules and rhizosphere soils. Different letters indicate significant differences between root nodules and rhizosphere soils of the three alder species (*p* < 0.05). *p < 0.05, **p < 0.01, and ***p < 0.001.

The one-way analysis of variance (ANOVA) results showed that the contents of total nitrogen (TN), ammonium nitrogen (AN) and nitrate nitrogen (NN) were significantly different between the root nodules and rhizosphere soils of the three alder species (Figure 1). The contents of TN and NN in the root nodules of *A. formosana* (AF_RN), *A. glutinosa* (AG_RN), and *A. cremastogyne* (AC_RN) were significantly higher than those of rhizosphere soils, while the content of AN was significantly lower than that of rhizosphere soils (Figure 1A). There were also significant differences in the TN and NN in the root nodules between the three alder species. The content of TN in AG_RN was significantly higher than that in AF_RN and AC_RN, and the content of NN in AF_RN was significantly higher than that in AG_RN and AC_RN. The AN content in AG_RN was the highest (1.44 mg/kg), 2.1 times that of the lowest (AF_RN) (Figure 1B). In the rhizosphere soil of the three alder species, there were significant differences in the contents between the three nitrogen types. The AN content in the rhizosphere soil of *A. formosana* (AF_S) was significantly higher than that in the rhizosphere soils of *A. glutinosa* (AG_S) and *A. cremastogyne* (AC_S). The content of TN in AF_S was the highest (0.79 g/kg), 6.1 times that of the lowest (AC_S). The highest content of NN was found in AG_S, which was 3.4 and 9.5 times that of AF_S and AC_S, respectively (Figure 1C).

### 3.2 Community characteristics of microorganisms in root nodules and rhizosphere soil

A total of 1,435,629 high-quality sequences (79,757 reads per sample) were obtained from all samples in 16S rRNA sequencing, with coverage above 99%. The probability of gene sequence detection in the samples was high, and the sequencing results accurately reflected the studied species. A total of 10,125 the same operational taxonomic unit (OTUs) were detected in all samples. There were considerable differences in the OTUs between the root nodules and rhizosphere soils of the three alders. The number of OTUs in AG_S was the highest (1,025) and 3.7 times that of the lowest (AC_RN). The number of OTUs in AF_RN and AG_RN was significantly (1.6 and 1.5 times) higher than that in AC_RN, respectively (Table 1). Microorganisms were identified in the root nodules and rhizosphere soils of the three alders by comparison with the SILVA database at an average of 15 phyla, 28 classes, 65 orders, 99 families, 137 genera, and 159 species. At the genus level, AG_RN and AG_S had the most actinobacteria taxa (144 and 198, respectively). In *nifH* gene sequencing, a total of 1,226,973 clean reads (68,165 clean reads per sample) were obtained from 18 samples. A total of 4,000 OTUs were detected in all samples. There were also considerable differences in the OTUs between the root nodules and rhizosphere soils, and the number of OTUs in the rhizosphere was higher than that in the root nodules (Table 1). Both in root nodules and in rhizosphere soils, AG_RN had the highest number of OTUs, which were 1.3 and 1.7 times higher in root nodules and 1.8 and 2.0 times higher in rhizosphere soil than those in AF_RN and AC_RN, respectively. A total of 34 orders, 58 families, 92 genera, and 134 species were identified in the root nodules and rhizosphere soil of the tree alders by comparison with the FunGene database. There were significant differences between the root nodules and rhizosphere soils at the family, genus, and species levels. However, there were no significant differences at the classification level in the root nodules and rhizosphere soils between the three alders.

**Table 1:** Composition of microorganisms in the root nodules and rhizosphere soils of different alder species.

	Group	Clean reads	OTUs	Phylum	Class	Order	Family	Genus	Species
16S rRNA	AF_RN	79,685 ± 167	438 ± 53	15 ± 2	26 ± 4	60 ± 14	88 ± 15	120 ± 10	144 ± 12
	AG_RN	79,844 ± 458	420 ± 61	16 ± 2	31 ± 3	69 ± 2	105 ± 2	144 ± 6	157 ± 7
	AC_RN	79,784 ± 308	274 ± 242	11 ± 6	18 ± 10	44 ± 24	65 ± 39	89 ± 60	100 ± 70
	AF_S	79,829 ± 138	634 ± 67	15 ± 2	30 ± 4	72 ± 11	112 ± 15	150 ± 20	178 ± 22
	AG_S	79,655 ± 250	1,025 ± 204	20 ± 2	38 ± 3	89 ± 9	136 ± 13	198 ± 8	230 ± 16
	AC_S	79,746 ± 207	584 ± 182	14 ± 2	25 ± 6	55 ± 15	85 ± 18	123 ± 34	148 ± 37
*nifH*	AF_RN	67,242 ± 2062	211 ± 43	5 ± 0	10 ± 0	19 ± 2	29 ± 3	35 ± 3	40 ± 5
	AG_RN	67,729 ± 1,673	275 ± 68	5 ± 1	11 ± 1	19 ± 6	27 ± 8	32 ± 11	43 ± 16
	AC_RN	66,043 ± 2022	157 ± 39	5 ± 0	10 ± 1	20 ± 2	29 ± 4	34 ± 5	40 ± 9
	AF_S	67,823 ± 1,014	338 ± 71	6 ± 0	10 ± 0	21 ± 1	34 ± 2	45 ± 3	55 ± 6
	AG_S	68,265 ± 672	599 ± 286	5 ± 0	11 ± 0	24 ± 1	35 ± 2	43 ± 4	55 ± 5
	AC_S	71,889 ± 10,359	295 ± 32	6 ± 0	12 ± 1	22 ± 2	33 ± 2	44 ± 2	56 ± 4

Data in the table are mean ± standard deviation (three biological replicates). AF_RN, AG_RN, and AC_RN represent the root nodules of *A. formosana*, *A. glutinosa*, and *A. cremastogyne*, and AF_S, AG_S, and AC_S represent the rhizosphere soils of *A. formosana*, *A. glutinosa*, and *A. cremastogyne*, respectively. The same applies below.

### 3.3 Diversity of microorganisms in root nodules and rhizosphere soil

In microorganism community by 16S rRNA, significant differences in the alpha diversity (ACE, Chao1, Simpson, and Shannon index) of the microorganism community were determined among different samples of the three alders (Table 2). The ACE and Chao1 indices in AG_S were significantly higher than those in AF_S and AC_S, indicating that the richness of AG_S was higher than that of AF_S and AC_S. The alpha diversity index of AG_S was significantly higher than that of AG_RN, indicating that the community diversity in AG_S was higher than that in AG_RN. In contrast, there were no significant differences in the alpha diversity of potential nitrogen-fixing microorganism by the *nifH* gene between the different samples of the three alders (Table 2). Nevertheless, the ACE, Chao1, and Shannon indices in rhizosphere soils were significantly higher than those in root nodules. Furthermore, the ACE, Chao1, and Shannon indices in AF_RN and AG_RN were higher than those in AC_RN. For instance, the ACE values in AF_RN and AG_RN were 1.4 and 1.8 times that in AC_RN. For rhizosphere soil N-fixing bacteria, the ACE and Chao1 values in AG_S were significantly higher than those in AF_S and AC_S. However, the highest Shannon and Simpson index values occurred in AF_S and were 1.5 and 1.6 times higher than the lowest value found in AG_S, respectively.

**Table 2:** Alpha diversity indices of microorganism communities in root nodules and rhizosphere soils of different alder species.

	Group	ACE	Chao1	Shannon	Simpson
16S rRNA	AF_RN	439.77 ± 54.19bc	438.28 ± 53.38bc	5.94 ± 0.94bc	0.91 ± 0.11a
	AG_RN	421.24 ± 60.96bc	420.13 ± 60.55bc	4.27 ± 1.45c	0.68 ± 0.18b
	AC_RN	275.16 ± 242.34c	274.44 ± 242.05c	4.71 ± 1.91c	0.88 ± 0.08a
	AF_S	636.04 ± 69.10b	634.02 ± 67.60b	7.18 ± 0.26ab	0.98 ± 0.00a
	AG_S	1027.67 ± 203.37a	1024.91 ± 203.70a	8.12 ± 0.61a	0.99 ± 0.01a
	AC_S	586.69 ± 181.94b	584.61 ± 181.63b	6.95 ± 0.65ab	0.97 ± 0.02a
	*F* value	8.462**	8.444**	4.68*	5.397**
*nifH*	AF_RN	216.83 ± 45.79	212.45 ± 43.29	4.39 ± 0.48	0.90 ± 0.04
	AG_RN	282.37 ± 68.29	277.43 ± 68.55	4.67 ± 0.78	0.89 ± 0.04
	AC_RN	157.33 ± 38.90	165.40 ± 40.47	2.67 ± 1.24	0.64 ± 0.20
	AF_S	343.51 ± 73.26	339.54 ± 71.39	5.53 ± 0.52	0.94 ± 0.02
	AG_S	606.57 ± 285.69	601.28 ± 285.14	3.57 ± 2.07	0.57 ± 0.30
	AC_S	306.74 ± 39.41	299.39 ± 35.18	4.49 ± 0.46	0.87 ± 0.04
	*F* value	2.942	2.964	1.625	2.073

Data in the table are mean ± standard deviation. * and ** represent significant differences at the levels of 0.05 and 0.01, respectively. Different lowercase letters in the same column indicate significant differences between root nodules and rhizosphere soils of different alders (*p* < 0.05).

**Figure 2:**
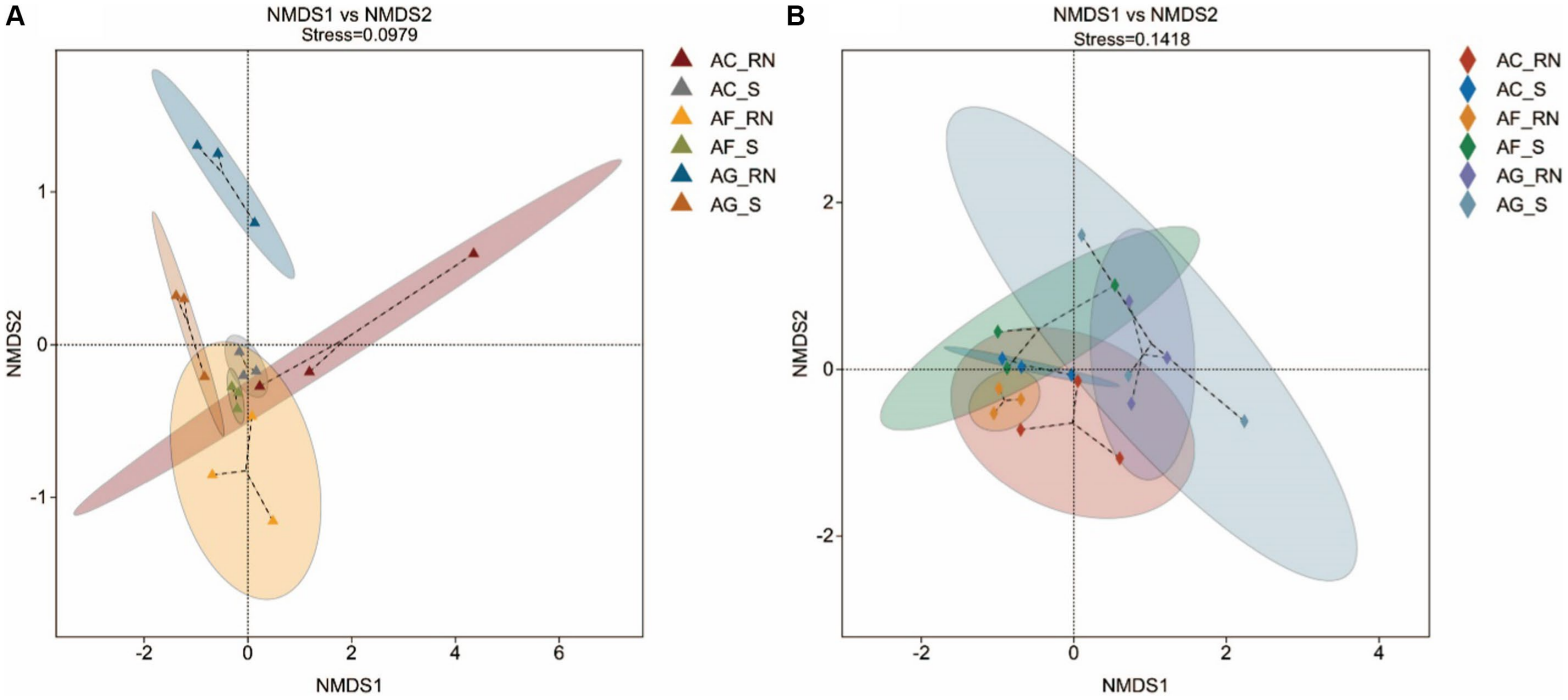
Nonmetric multidimensional scaling (NMDS) analysis of microorganisms by 16S rRNA **(A)** and *nifH* gene **(B)** in the root nodules and rhizosphere soils of three alder species. When the stress is less than 0.2, NMDS analysis is considered robust. The closer of the samples are on the coordinate diagram, which means the higher of their similarity is.

The nonmetric multidimensional scaling (NMDS) results of the 16S rRNA and *nifH* genes both showed that the distance between the root nodules was larger, and the distance between the rhizosphere soils was smaller, indicating that the microorganism communities were greatly different between the root nodules of the three alders (Figure 2). The results of the *nifH* gene showed that the distance between the root nodules of the three alders was larger than the distance among the rhizosphere soils, which also suggests that the communities of potential nitrogen-fixing microorganism between the root nodules of the three alders were greatly different. The differences in microorganisms between the root nodules and rhizosphere soils were small in *A. formosana* and large in *A. glutinosa*, suggesting a great difference in microorganism communities between the root nodules and rhizosphere soil of *A. glutinosa*.

### 3.4 Comparative classification analysis of microorganisms among the three alders

**Figure 3:**
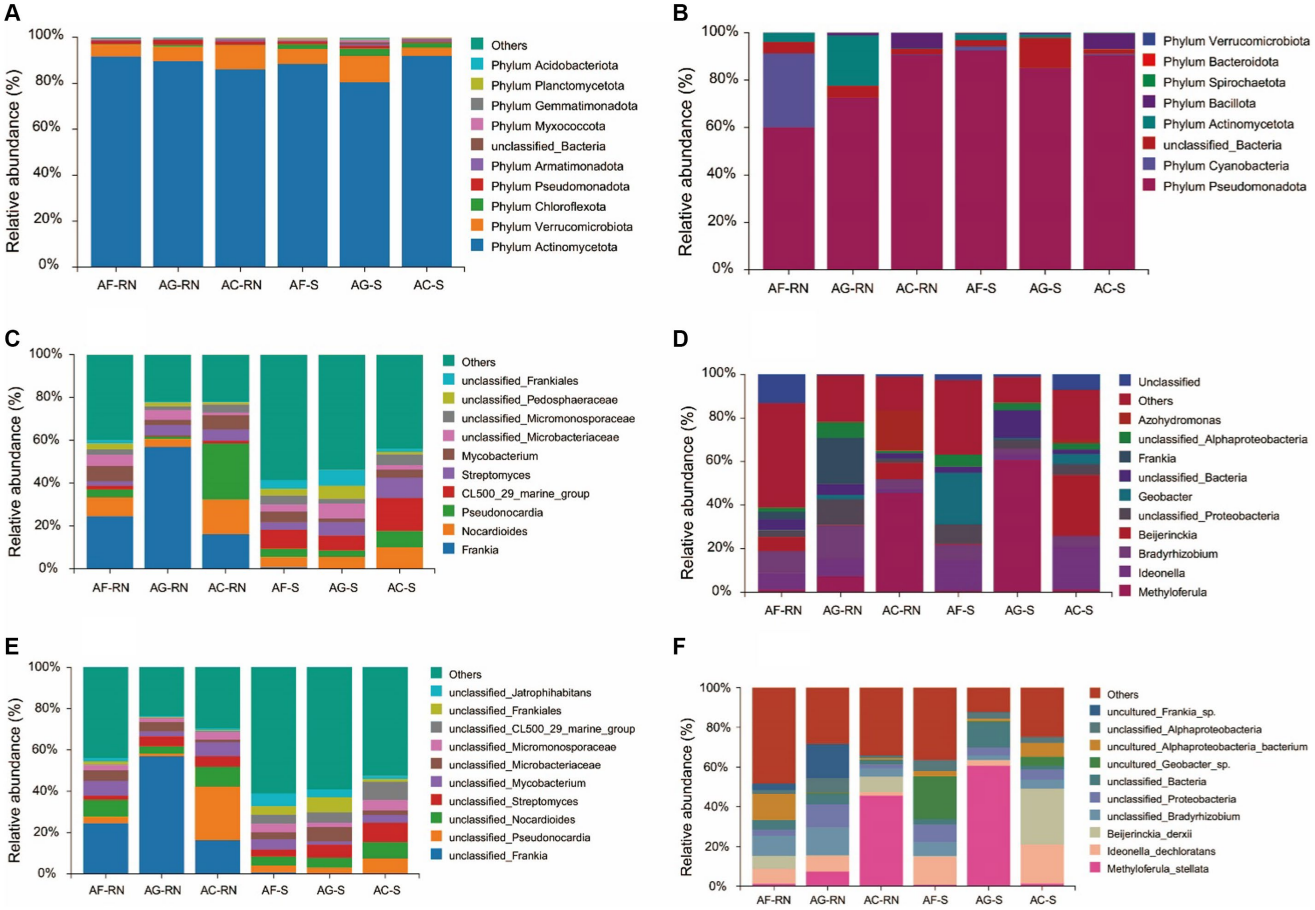
Relative abundance of the top 10 microorganism communities at the phylum, genus, and species levels. **(A–C)** Relative abundance of microorganism communities by 16S analysis at the phylum, genus, and species levels, respectively. **(D–F)** Relative abundance of microorganism communities by *nifH* analysis at the phylum, genus, and species levels, respectively.

The comparative classification results of 16S rRNA analysis showed that the dominant phyla in the root nodules of alders were phylum Actinomycetota (86.08–91.70%), phylum Verrucomicrobiota (5.16–10.58%) and phylum Pseudomonadota (1.45–2.12%). At the phylum level, the dominant phyla in the rhizosphere soils were phylum Actinomycetota (80.41 ~ 91.88%), phylum Verrucomicrobiota (3.65 ~ 11.65%) and phylum Chloroflexota (2.05 ~ 3.06%) (Figure 3A). For the top three dominant phyla with the highest relative abundances in the root nodules, the samples with the maximum relative abundance of each bacterium are shown below: The relative abundances of phylum Actinomycetota (91.70%), phylum Verrucomicrobiota (10.58%) and phylum Pseudomonadota (2.12%) were the highest in AF_RN, AC_RN and AG_RN, respectively. In the rhizosphere soils, phylum Actinomycetota (91.88%) had the highest relative abundance in AC_S, and phylum Verrucomicrobiota (11.65%) and phylum Chloroflexota (3.06%) had the highest relative abundance in AG_S. At the genus level, except for *A. cremastogyne*, *Frankia* (16.17–56.90%) was the dominant genus in the root nodules (Figure 3B). *Frankia* was the dominant genus in AF_RN (24.54%) and AG_RN (56.90%), while *Pseudonocardia* (26.21%) was the dominant genus in AC_RN. The dominant genus in the rhizosphere soils of *A. formosana* and *A. cremastogyne* was CL500_29_marine_group (6.97 ~ 15.43%), and CL500_29_marine_group was the dominant bacteria in AF_S (8.85%) and AC_S (15.43%). Unclassified_*Frankia* was the dominant genus in AG_S (7.35%). The relative abundance of *Frankia* in root nodules was higher than that in the rhizosphere soils, but the relative abundance of unclassified_*Frankia* was lower than that in the rhizosphere soils. At the species level, unclassified_*Frankia* was the superior species in the root nodules of *A. glutinosa* and *A. formosana*, with the same ranking of relative abundance as *Frankia* (16.17 ~ 56.90%) (Figure 3C). In AF_RN and AG_RN, the dominant bacteria with the highest relative abundance was unclassified_*Frankia*, with 24.54 and 56.90%, respectively, and the dominant species in AC_RN was unclassified_Pseudonocardia (26.13%). The dominant species in the rhizosphere soils of the three alder species were different. The relative abundance of unclassified_*Frankia* in the root nodules of the three alder species was greater than that in the rhizosphere soils.

The *nifH* gene results showed that the dominant phyla of alders were phylum Pseudomonadota (60.12–90.67% in root nodules and 85.05–92.57% in the rhizosphere), phylum Cyanobacteria (0.03–31.17% in root nodules and 0.06–1.63% in the rhizosphere), unclassified bacteria (2.37–4.82% in root nodules and 1.90–12.72% in the rhizosphere), and phylum Actinomycetota (0.16–21.2% in root nodules and 0.09–2.56% in the rhizosphere) (Figure 3D). In root nodules, the relative abundance of phylum Pseudomonadota in *A. cremastogyne* (90.67%) was significantly higher than that in *A. glutinosa* (72.61%) and *A. formosana* (60.12%); however, the relative abundance of phylum Cyanobacteria in *A. formosana* (31.17%) was significantly higher than that in *A. glutinosa* (0.03%) and *A. cremastogyne* (0.20%). *Bradyrhizobium*, *Frankia*, and *Methyloferula* were the dominant genera of potential nitrogen-fixing microorganism in AF_RN (10.02%), AG_RN (21.19%), and AC_RN (45.60%), respectively. In rhizosphere soils, *Geobacter*, *Methyloferula*, and *Beijerinckia* were the dominant genera in AF_S (23.61%), AG_S (60.72%), and AC_S (27.95%) (Figure 3E). At the species level, the top dominant species in AF_RN, AG_RN, and AC_RN were uncultured_Alphaproteobacteria_bacterium, uncultured_Frankia_sp., and Beijerinckia_derxii, respectively, and the top dominant species in AF_S, AG_S, and AC_S were uncultured_Geobacter_sp., Methyloferula_stellata, and Beijerinckia_derxii, respectively (Figure 3F). There were significant differences in the dominant species of potential nitrogen-fixing microorganism among the root nodules and rhizosphere soil between the three alders. For instance, the relative abundance of uncultured_Alphaproteobacteria_bacterium in AF_RN was 45.4 and 13.7 times that in AG_RN and AC_RN, respectively, and the relative abundance of Methyloferula_stellata in AG_S was 75.9 and 44.6 times that in AF_S and AC_S, respectively.

### 3.5 Intergroup difference analysis of microorganism

**Figure 4:**
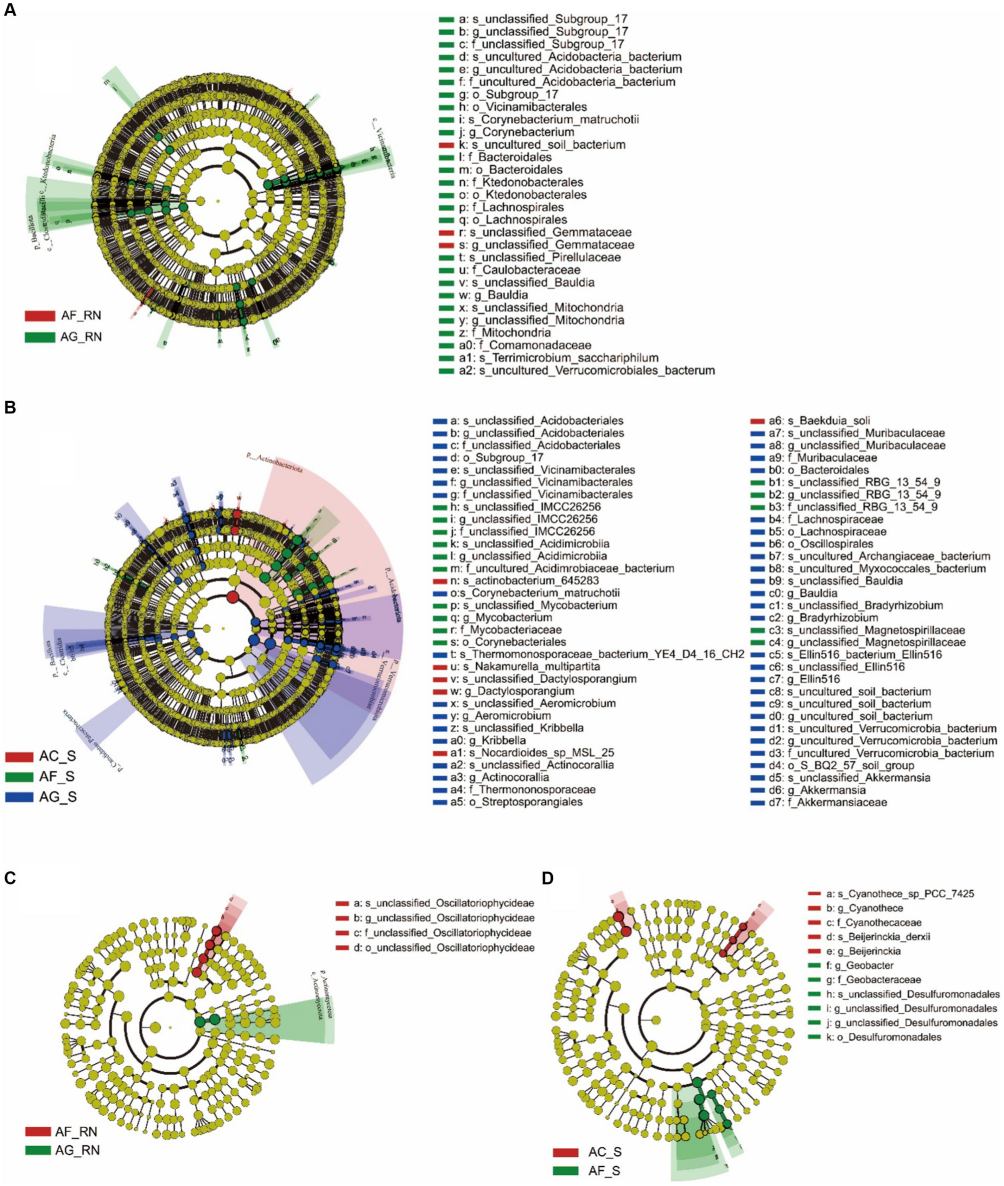
LEfSe analysis of the microorganism communities in root nodules and rhizosphere soil of three alder species. **(A,B)** LEfSe analysis of the microorganism community in root nodules and rhizosphere soils according to 16S rRNA data. **(C,D)** LEfSe analysis of the microorganism community in root nodules and rhizosphere soils according to the *nifH* gene. The circle represents the phylogenetic level from phylum to species (from inner circle to outer circle). The diameter of each circle is proportional to the abundance of the population. The term “uncultured” refers to an unidentified species obtained directly from the database by sequence comparison.

LEfSe analysis of microorganisms from 16S rRNA data showed that there were three groups of microorganisms significantly enriched in AF_RN and AG_RN (Figure 4A): *A. formosana*: unclassified_*Gemmataceae* (from genus to species), *A. glutinosa*: Vicinamibacteria (order) and Ktedonobacteria (from order to family). In the rhizosphere soils of the three alder species, 14 groups of microorganisms were significantly enriched (Figure 4B). The dominant groups in *A. formosana* were unclassified_RBG_13_54_9 (from family to species), Mycobacteriaceae (from family to species), Corynebacteriales (order), unclassified_Acidimicrobiia (from family to species) and unclassified_IMCC26256 (from family to species). The dominant groups in *A. glutinosa* were unclassified_Vicinamibacterales (from family to species), *Actinocorallia* (from genus to species), Bacteroidales (order), Bacillota (phylum), Clostridia (class), Candidatus Patescibacteria (phylum), Verrucomicrobia (from phylum to class), and Acidobacteriota (phylum). The superior group in *A. cremastogyne* was *Dactylosporangium* (from genus to species). These results indicated that the dominant groups differed significantly between the three alders in the root nodules and rhizosphere soils, except for AC_RN.

LEfSe analysis of potential nitrogen-fixing microorganism from *nifH* gene data showed that there were two groups of microorganisms significantly enriched in AF_RN and AG_RN: *A. glutinosa*: Actinomycetota (from phylum to class) and *A. formosana*: un_classified_Oscillatoriophycideae (from order to species) (Figure 4C). In the rhizosphere soils, nine groups of microorganisms were significantly enriched (Figure 4D). The dominant groups in *A. cremastogyne* were Cyanothece_sp_PCC_7425 (family), *Cyanothece* (genus), *Beijerinckia_derxii* (genus), *Cyanothecaceae* (species), and *Beijerinckia* (species). The dominant groups in *A. formosana* were unclassified_Desulfuromonadales (from family to species), Geobacter (order), Geobacteraceae (family), and *Desulfuromonadales* (genus). These results suggested that the dominant groups differed significantly between different alders.

### 3.6 Functional predictions and differential analysis of microorganisms by 16S rRNA analysis

**Figure 5:**
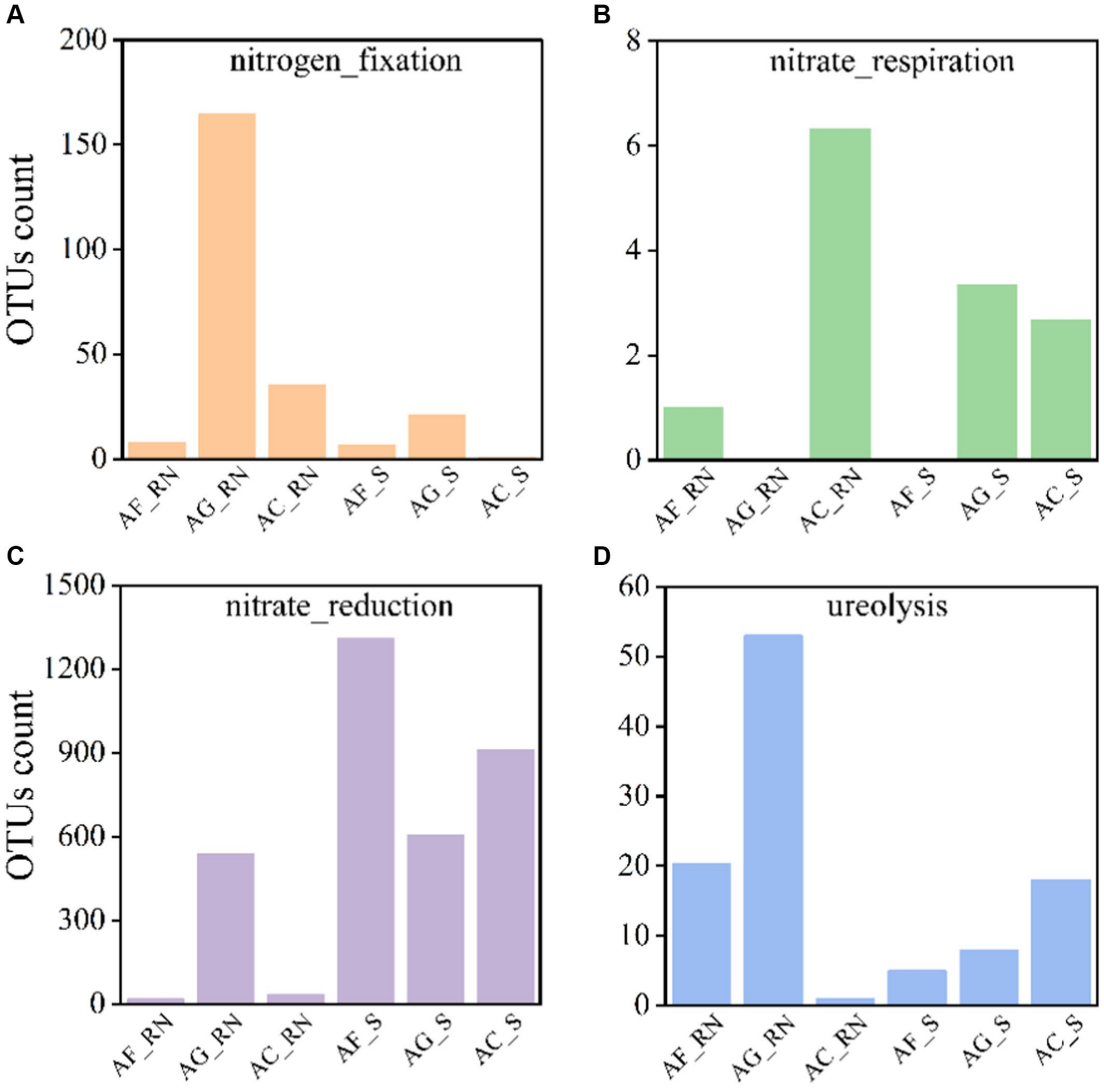
Number of actinomycetes OTUs associated with N cycle functions in root nodules and rhizosphere soils of three alder species.

The microorganism had similar functional structures in the root nodules and rhizosphere soils. In all root nodule and rhizosphere soil samples for the three alders, the microorganisms associated with anaerobic chemoheterotrophy had the largest average number of OTUs (11,271 (AG_RN, minimum)-29,181 (AC_RN, maximum), average = 18,680), followed by aerobic chemoheterotrophy (11,222 (AG_RN)-29,162 (AC_RN), average = 18,634) and aromatic compound degradation (1,947 (AG_RN)-7,761 (AC_RN), average = 3,748) (Supplementary Table S1). To further understand the differences between the potential nitrogen (N) functions, the N cycle function was predicted (Figure 5). Four N cycle functions were mostly noted: Nitrogen fixation, nitrate respiration, nitrate reduction, and ureolysis. The number of OTUs associated with N fixation in AG_RN was higher than that in AF_RN and AC_RN (Figure 5A). The number of OTUs with nitrate respiration in AC_RN was the highest, with an average of 6.33, which was 6.33 times higher than that of AF_RN (Figure 5B). Except for the number of OTUs with ureolysis function in AC_RN being smaller than that in AC_S, the number of OTUs with N fixation and ureolysis functions in the root nodules of the three alder species was higher than that in rhizosphere soils, but the number of OTUs with nitrate reduction function was lower than that in rhizosphere soils.

### 3.7 Relationships between N nutrients and microorganisms

**Figure 6:**
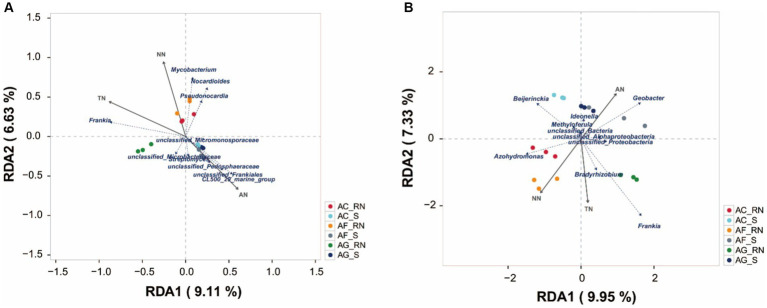
Redundancy analysis of microorganisms of three alder species at the genus level according to 16S rRNA data **(A)** and the *nifH* gene **(B)**.

Redundancy analysis (RDA) revealed that the characteristics of N nutrients explained 14.68 and 17.28% of the total variation in the microorganism communities (Figure 6). The content of TN had the greatest influence on the actinobacteria communities followed by that of NN and AN (Figure 6A). The contents of TN and NN influenced the actinobacteria community in the root nodules of the three alders, and the content of AN influenced the actinobacteria communities in the rhizosphere soils. The contents of TN and NN were positively correlated with *Frankia* and *Mycobacterium*, indicating that they affect the communities of *Frankia* and *Mycobacterium*. NN was positively associated with unclassified_*Pedosphaeraceae*, unclassified_*Frankia*, CL500_29_marine_group, and *Streptomyces*, indicating that they mainly affected the communities of these microorganisms. The contents of N nutrients also greatly influenced potential nitrogen-fixing microorganism communities (Figure 6B). The AN content positively influenced *Geobacter* in rhizosphere soils but the NN content negatively influenced on *Geobacter* in root nodules. The content of TN was positively correlated with *Frankia*, but negatively influenced *Beijerinckia*. *Azohydromonas* was positively associated with NN, but negatively associated with AN.

## 4 Discussion

Actinorhizal plants are woody nonleguminous plants characterized by their ability to form root nodules in symbiosis with the nitrogen-fixing actinobacterium *Frankia* (Vemulapally et al., 2022b). *Alnus* spp. (alder) and mycorrhizae have a symbiotic relationship that helps alder take up nitrogen (N) nutrients, while actinorhizal symbiosis provides assimilable N. It is through these efficient symbiotic relationships that actinorhizal plants, such as alder, can colonize poor substrates, enrich the soil, and initiate plant succession (Roy et al., 2007). The most restricting nutrient for plant productivity is N (Vitousek and Howarth, 1991). Most plants mainly rely on inorganic N in the soil solution because they cannot directly utilize macromolecular organic N in the soil (Jones et al., 2005). The results showed that the contents of total nitrogen (TN) and nitrate nitrogen (NN) in the root nodules of the three alder species were significantly greater than those in the rhizosphere soils, while the ammonium nitrogen (AN) content in root nodules was significantly lower than that in rhizosphere soils. In *Avena barbata*, the total rate of N mineralization in rhizosphere soil is approximately ten times higher than that in bulk soil, and the interaction between microorganisms and roots may accelerate the conversion of organic N into plant-available AN (Herman et al., 2006). Alternatively, increases in microbial numbers and activity associated with root carbon (C) may attract bacterivores, which consume low C/N microbial biomass and release N as AN into the rhizosphere.

Actinorhizal plants harbor similar non-*Frankia* plant growth-promoting-bacteria as legumes and other plants, and the prevalence of *Frankia* in the root nodule is influenced by environment, species, genotypes, and growth stages (Ghodhbane-Gtari et al., 2021; Sohn et al., 2021). In the root nodules and rhizosphere soils, the alpha diversity of microorganisms in *A. glutinosa* was significantly higher than that in *A. cremastogyne* and *A. formosana*, indicating that the community abundance and diversity of nitrogen fixation related bacteria are greater in *A. glutinosa*. Nonmetric multidimensional scaling (NMDS) analysis also illustrated greater differences in microorganisms between the root nodules of the three alders, although the differences in potential nitrogen-fixing microorganism by the *nifH* gene were lower than those in actinobacteria by 16S rRNA sequencing because the formation of actinorhizal root nodules is the result of the combined action between the plant genotype, *Frankia* genotype and environment (Chaia et al., 2010). The classification results indicated that the dominant phyla in the root nodules and rhizosphere soil of the three alders was phylum Actinomycetota, which was similar to the results for the nonleguminous species sea buckthorn (*Hippophae rhamnoides* L.) and actinorhizal species *A. cremastogyne* (Liu et al., 2022; Keyao et al., 2023). The biogeographic patterns and assembly process of the rhizobium communities differed in the rood nodule and the rhizosphere soil, which derived the significant differences in bacterial community composition in the root nodules and rhizosphere soils (Jing et al., 2022). In our study, there were obvious differences in the dominant groups between root nodules and rhizosphere soils. For instance, phylum Actinomycetota and phylum Verrucomicrobiota showed similar relative abundances in the root nodules and rhizosphere soils, while phylum Pseudomonadota and other microorganisms showed higher relative abundances in the root nodules than in the rhizosphere soils. These results suggested that the host selectively shaped the structure and abundance of endophytic bacterial communities in the root nodules and rhizosphere soils (Keyao et al., 2023). This can also be explained by the niche theory. For instance, soybean (*Glycine max* (L.) Merr.) select rhizosphere microbial communities based on functional traits, which may be related to growth promotion and nutritional benefits for plants. These results reflected a plant’s selective ability to shape microbial communities at the classification and functional levels (Mendes et al., 2014). A previous study showed that Pseudomonadota was one of the dominant phylum in *A. cremastogyne* monocultures and mixed plantations (Liu et al., 2022). The phylum Pseudomonadota was also the dominant phyla in the root nodules of the three alders, which is similar to the findings that phylum Pseudomonadota is the main nitrogen-fixing group in the forest ecosystem (Izquierdo and Nüsslein, 2006). In addition, phylum Pseudomonadota belongs to the group of autogenous nitrogen-fixing bacteria among nonsymbiotic nitrogen-fixing bacteria. Although nonsymbiotic nitrogen-fixing bacteria have a low N fixation rate, they are widely distributed in various ecosystems (Elbert et al., 2012). At present, rhizobia have been found in root nodules of many legumes, such as *Astragalus* L. (Lei et al., 2014), co-occur with a variety of nonsymbiotic nitrogen-fixing microorganisms. We showed that the nonlegume alder also has nonsymbiotic nitrogen-fixing endophytes in the root nodule. Numerous studies have shown host specificity for the community composition of endophytic bacteria (Wearn et al., 2012), which is primarily influenced by the species, function, and tissue of the host (Laforest-Lapointe et al., 2017). The actinobacteria of root nodules and rhizosphere soils of the three alders differed in their community structure and composition, demonstrating the host specificity of the actinobacteria. During N fixation, the number of actinobacteria the same operational taxonomic unit (OTU) in the root nodules of *A. glutinosa* was higher than that of *A. formosana* and *A. cremastogyne*. In the root nodules of *A. glutinosa*, there were additional microorganisms with nitrogenases. These results indicated that the nitrogen fixation capacity of *A. glutinosa* would be better than that of other two alders. Limnohabitans have been found to contain nitrite reductase and urease in freshwater habitats, which function the N cycle, such as in nitrite reduction and ureolysis (Zeng et al., 2012). Additionally, the number of OTUs in the root nodules of the three alder species was higher than that in the rhizosphere soils, while the number of OTUs with nitrate reduction function in the root nodules of the three alder species was lower than that in the rhizosphere soils. The above results suggested that the number of microorganisms with nitrogenases in the root nodules of the alder trees is higher than that in the rhizosphere soil, while the number of actinobacteria with nitrate reductases is lower than that in the rhizosphere soil. Therefore, due to the varying diversity of actinobacteria in the three alders, as well as the microorganisms’ different functional enzymes, the N cycle functions are different between the hosts and between the root nodules and the rhizosphere soils.

Nonleguminous plants that form root nodules after being infected with *Frankia*, a gram-positive actinobacteria, are collectively known as actinorhizal plants (Lechevalier, 1994; Benson et al., 2004; Ardley and Sprent, 2021). *Alnus* spp. is the most widely distributed actinorhizal plant genus that associates with the *Frankia alni* species complex (Põlme et al., 2014). *Frankia* inhabits important ecological niches, such as root nodules that are symbiotic with a variety of woody plants (Samant et al., 2016). In contrast with the classification results of 16S rRNA sequencing, *nifH* gene data showed that the dominant phyla in the root nodules and rhizosphere soils was phylum Pseudomonadota, and there was a significant difference in the dominant microorganisms in different alders, suggesting that there were plentiful N fixation-related bacteria in alder roots in addition to *Frankia*. Soil environmental conditions and host plant genotype both affect the selection of *Frankia* strains by a host plant for root nodule formation (Pokharel et al., 2011). Several studies have suggested that different plants type and genotypes of the same plant species harbor partially different microbiomes (Berlanas et al., 2019). We also found that the root nodules from three alders growing on the same soils demonstrated the presence of different *Frankia* populations, indicating that the host plant genotype significantly affected on the occurrence of *Frankia* strains. For members of the *Alnus* spp. host infection group, differences in the abundance of nodules were found as a function of host plant species, with nodule numbers consistently being greatest on *A. rubra*, and lower on *A. incana* subsp. *incana*, and lowest on *A. glutinosa* (Huss-Danell and Myrold, 1994). However, in this study, the relative abundance of *Frankia* in the root nodules of *A. glutinosa* was significantly higher than that in *A. cremastogyne* and *A. formosana*. It has been speculated that the presence of a *Frankia* strain in nodules is positively related to its abundance in the soil (Dai et al., 2004). Our results supported this speculation because the relative abundance of *Frankia* by *nifH* gene analysis in root nodules and rhizosphere soils followed a consistent order: *A. glutinosa* > *A. formosana* > *A. cremastogyne*. Pokharel et al. (2011) contradicted this speculation because *nifH* gene clone library analysis retrieved only sequences representing *Frankia* distantly related to those in nodules, with sequences that were least abundant in nodules being the most similar to those from soil (Pokharel et al., 2011). However, similar results to our *nifH* gene analysis were also obtained from 16S rRNA data. Thus, we consider that the *Frankia* strains in nodules can affect the abundance of those in soil.

Nitrogen-fixing bacteria can produce substances that help plants grow, and they can also provide nonleguminous plants with a large amount of N, which increases the availability of additional nutrients (phosphorus, kalium, and zinc) (Aasfar et al., 2021). Thus, rhizobia can grow using host plants of organic compounds for their carbon, nitrogen, and energy requirements. Root nodule bacteria require access to adequate concentrations of nutrients (e.g., nitrogen, carbon, and oxygen) for metabolic processes to enable their survival and growth as free-living soil saprophytes, and in their symbiotic relationship with legumes (O'hara, 2001). In pine forest, N fertilization strongly affects the *nifH* community structure (Berthrong et al., 2014). In this study, the results of the RDA showed that the contents of TN and NN positively influence on *Frankia* community. It can be speculated that TN and NN are associated with the diversity of the *Frankia* community in alders, and non-*Frankia* actinobacteria, such as *Mycobacterium* and *Bradyrhizobium*, and TN was also positively correlated with the diversity of *Mycobacterium* and *Bradyrhizobium*. These results confirm the influence of *Frankia* strains on the N nutrients in each alder species. While these results provide novel information on the effect of N nutrients on nodules, additional studies will be needed to resolve the issues concerning the abundance and occurrence of infectious *Frankia* particles in soil.

## 5 Conclusion

In this study, we determined the differences in the contents of nitrogen (N) nutrients and in the community structure and diversity of microorganisms in root nodules and rhizospheresoils by 16S rRNA and *nifH* gene sequencing between three *Alnus* spp. The contents of total nitrogen (TN) and nitrate nitrogen (NN) in the root nodules of the three alder species are significantly higher than those in the rhizosphere soils, while the content of ammonium nitrogen (AN) is significantly lower in the root nodules than in the rhizosphere soils. The diversity of the microorganism communities in the root nodules and rhizosphere soil of *A. glutinosa* is greater than those in *A. formosana* and *A. cremastogyne*. Additionally, the root nodules of the three alders have higher numbers of OTUs with N fixation functions than the rhizosphere soils. The relative abundances of *Frankia* in *A. glutinosa* root nodules and rhizosphere soils are significantly higher than those in *A. cremastogyne* and *A. formosana*. The results of the redundancy analysis (RDA) showed that the TN content had the largest impact on the relative abundance of the *Frankia* community compared to the other bacterial communities. TN and NN are positively associated with *Frankia*. Therefore, we speculate that the N fixation ability of root nodules is greater than that of rhizosphere soils, and *A. glutinosa* has a stronger N fixation ability than *A. formosana* and *A. cremastogyne*. These findings provide new information about the community structure and N-fixing ability of potential nitrogen-fixing microorganisms in different alder species and serve as a reference for applying *Frankia* in alder plantations.

## Data availability statement

The datasets presented in this study can be found in online repositories. The names of the repository/repositories and accession number(s) can be found at: BioProject, PRJNA982722.

## Author contributions

HG and HY: conceptualization and validation. YY: methodology and writing—original draft preparation. ZC and HY: software. YY and XH: formal analysis. FW and ZH: investigation. ZC: resources. HG and ZH: data curation. HY: writing—review and editing. HG: visualization. All authors have read and agreed to the published version of the manuscript.
